# Validation of a patient-administered questionnaire to measure the activity impairment experienced by women with uncomplicated urinary tract infection: the Activity Impairment Assessment (AIA)

**DOI:** 10.1186/1477-7525-3-42

**Published:** 2005-07-15

**Authors:** Diane J Wild, Darren J Clayson, Karen Keating, Kathleen Gondek

**Affiliations:** 1 Oxford Outcomes Ltd, Old Barn, Jericho Farm, Cassington, Oxford, OX29 4SZ, UK; 2 Bayer Healthcare Pharmaceuticals, 400 Morgan Lane, West Haven, CT 06515-4175, USA

## Abstract

**Background:**

To validate a questionnaire to assess the activity impairment associated with uncomplicated urinary tract infection (uUTI).

**Methods:**

The Activity Impairment Assessment (AIA) assesses the amount of time an individual's work or regular activities have been impaired as a result of their UTI. The measure was completed by 276 women with uUTI who had participated in a prospective, open-label, non-comparative multi-centre clinical trial of CIPRO^® ^XR (extended-release ciprofloxacin). Baseline scores on the King's Health Questionnaire (KHQ) and clinical symptom evaluations were collected for validation purposes.

**Results:**

An exploratory factor analysis showed that all items loaded >0.84 on a single component. This uni-dimensional structure was supported by Rasch analysis. The AIA was found to have excellent levels of internal consistency (Cronbach's alpha = 0.93), convergent validity (all r_s _>.70) and divergent validity (r_s _= .078). The AIA displayed excellent discriminant validity in relation to clinical evaluations, and was found to be responsive to change across all clinical evaluations.

**Conclusion:**

The unidimensional AIA shows high levels of internal reliability, convergent and divergent validity, discriminant validity and responsiveness. It is an excellent tool for measuring activity impairment in UTI.

## Background

Urinary tract infections (UTIs) are one of the most common conditions seen by general practitioners, representing a significant healthcare cost burden [1]. In the USA alone there are an estimated seven million physician visits and one million emergency department visits each year specifically for UTIs [2,3]. Although it has been reported that half of all women experience at least one UTI in their lifetime [2,3], the incidence of UTI is difficult to assess accurately because UTI is not well reported.

UTIs may be either 'uncomplicated' or 'complicated'. Complicated UTIs are associated with metabolic, functional, or anatomic abnormalities. An acute uncomplicated UTI (uUTI) (also referred to as cystitis, acute cystitis, and dysuria-frequency syndrome) has been defined in a number of ways. In women, it includes a clinical syndrome characterised by various combinations of dysuria (painful urination), frequency, urgency, gross haematuria, lower back and/or abdominal/suprapubic discomfort with pyuria and bacteriuria [4,5]. As many as half of all patients with uUTI may not have bacteriuria according to established criteria, and have no known underlying renal or urologic dysfunction or obstruction [6].

While uUTI is generally considered a benign condition, it is associated with significant short-term morbidity. The nature and extent of these limitations has led to this benign classification being questioned [7]. Malterud and Baerheim [8] recently demonstrated a wide range of different symptoms in patients with uUTI. The authors concluded that traditional medical knowledge about symptom presentation in uUTI is insufficient, and that awareness of symptom diversity and highly individual presentation may lead to better recognition of the atypical patient, and to improved diagnosis, treatment, and care [9].

While there has been some research on the impact of uUTI on everyday activities, little is known about the impact of UTI on everyday routine. Foxman and Frerichs [10] reported that each episode of UTI results in an average of 6.1 symptomatic days and 2.4 restricted-activity days, as well as time lost from work. In a recent Canadian survey [11], almost half (48%) of Canadian women who had experienced a UTI reported that it affected at least one of their daily activities. Additionally, Decima Research Incorporated (2002) reported on the modification of the Williamson Functional Impairment Scale to measure outcomes of impairment due to UTI, such as time lost from usual daily activities.

The Activity Impairment Assessment (AIA) was developed for the purposes of the present study by the authors in 2003 and was based on an existing work productivity measure (the Stanford Presenteeism Scale (SPS-6) [12] to evaluate the impact of health problems on individual performance and productivity. For the AIA measure, the concept of work was broadened to include other activities and social activities. The AIA includes 5 items, the content of which can be seen in table 1. The present study was designed to validate the AIA in the assessment of activity impairment associated with uUTI.

**Table 1:** Distribution of AIA responses to each individual item

**AIA items and total score**	**Mean (SD)**	**Median**	**Range**
Cut down on time at work	1.52 (1.07)	2	0–4
Accomplished less	1.67 (1.10)	2	0–4
Limited in kind of work	1.44 (1.20)	2	0–4
Difficulty performing work	1.55 (1.20)	2	0–4
Interfered with social activity	1.38 (1.29)	2	0–4
Total score	7.56 (5.22)	2	0–4

The data for the validation study came from a clinical trial of the effectiveness of CIPRO^® ^XR (extended-release ciprofloxacin) in the treatment of uUTI. The trial was designed to assess time to improvement of symptoms, the size of any improvements, and the extent to which women can resume their daily activities following treatment. The psychometric properties of the AIA were assessed primarily by the pattern of associations between AIA scores and scores on the validated King's Health Questionnaire (KHQ). The assessed properties included internal consistency, convergent and divergent reliability, discriminant validity, and responsiveness.

## Methods

### Assessment of reliability and validity

Reliability and validity assessment was conducted in the context of a 3-day prospective, open-label, non-comparative, multi-centre clinical trial of CIPRO^® ^XR 500 mg. Women with uUTI were recruited for entry to this trial between June 18^th ^2003 and January 23^rd ^2004. At first visit, prior to receiving the first dose of study medication, patients gave written informed consent, a urine sample for dipstick biochemical analysis for nitrites or leukocyte esterase, and a clear-catch midstream urine specimen for culture and sensitivity. Patients provided demographic and medical history details (age, ethnicity, years of education, employment status, previous history of uUTI, number of days since onset of uUTI before seeing physician) and completed the patient-reported questionnaires.

Patient reported outcomes were recorded in electronic format using PalmOS™ palm pilots, with information transferred to a host computer.

### Activity Impairment Assessment (AIA)

The AIA is a self-administered 5-item questionnaire assessing the amount of time, over the previous 24 hours, that an individual's work or regular activities have been impaired as a result of their UTI. Patients respond to AIA items on a 5-point Likert-type scale, with the response options 'none of the time', 'a little of the time', 'some of the time', 'most of the time' and 'all of the time', scored 0–4.

The AIA was administered on day 2, then every 24 hours until regular daily activities had been unimpaired by UTI for 24 hours. Administration was stopped after the patient indicated at two consecutive administrations of the questionnaire (over at least 24 hours) that she had no activity impairment. The measure was completed at Test of Cure (TOC) visit if the UTI symptoms had persisted or if regular daily activities were impaired.

### King's Health Questionnaire (KHQ)

The KHQ is a self-administered questionnaire designed to assess the impact of urinary incontinence on QoL in women. The measure contains 21 questions that are scored in nine domains (general health perception, incontinence impact, role limitations, physical limitations, social limitations, personal relationships, emotions, sleep/energy, severity of urinary symptoms) [13]. Weighted summary scores in each domain range from 0 to 100, with higher scores indicating greater impairment. Part III of the questionnaire is a list of 10 bladder problems plus an 'other' category. These items are not summed to form a domain score.

The KHQ was chosen as the primary instrument in the validation of the AIA because it has been validated for use in the assessment of women with urinary problems [14,15] and it contains questions on "bladder problems" (i.e., frequency, urgency, bladder pain etc.) and how much these problems presently affect the subject.

The KHQ was administered at day 1, day 3, when the subject indicated that the UTI symptoms had resolved (no symptoms for 24 hours, or over three data capture points, whichever was longer), and at the TOC visit.

Clinical evaluation involved an assessment of five UTI symptoms (dysuria, frequency, urgency, suprapubic pain, gross haematuria) rated 'none', 'mild', 'moderate', or 'severe', and scored 0–3. The clinical evaluations took place at visit 1, at any premature discontinuation (day 1 to day 3), and at the TOC visit.

### Statistical methods

The data were analysed primarily in SPSS version 12.0 [16]. The Normality of the distributions of the AIA and KHQ scores was assessed both visually (using the histogram and superimposed Normal curve) and numerically using the relation between the distribution mean and the standard deviation and Kolmogorov-Smirnov statistics. Where data were found to be non-normally distributed, non-parametric tests were used. Analysis of variance with post-hoc Tukey tests (or non-parametric Kruskal-Wallis tests with Bonferroni-corrected post-hoc Mann-Whitney tests) were used to compare group scores. Associations between two continuous variables (absolute or change scores) were assessed using Pearson's r, (or non-parametric Spearman's r_s_) correlation coefficients. Missing values were handled by excluding cases pairwise. Examination of the dimensionality of the AIA, and the functioning and fit of individual items, was undertaken, as described below, by fitting a Rasch unidimensional measurement model in RUMM2010 [17]. Statistical significance throughout was taken at the 5% level (P < .05).

### AIA factor structure

Exploratory factor analysis using principle components extraction and varimax rotation was performed on the 5 AIA items to explore the structure of the measure. Factors were extracted if their eigenvalue was >1. Domain scores of any resulting factors, or of a total score, were calculated as a sum of the component item scores.

### Item functioning

Because the AIA does not directly assess symptoms, it was appropriate to explore the performance of the AIA with the use of item response theory (IRT). IRT allows an understanding of how people respond to items and how items measure an underlying dimension [18]. One of the simplest IRT models is the one-parameter Rasch model. The Rasch model assumes that the probability of a respondent giving a 'correct' answer (or responding at a given level on the scale) to a particular item is a logistic function of the relative distance between the item location parameter and the respondent location parameter [19]. The item-trait interaction Χ^2 ^statistic was used to assess the fit of the data to the model, and the item-person interaction mean and SD values (where perfect fit is indicated by a mean of zero and SD of 1.0) to assess the degree of consensus displayed collectively by all items of the instrument across persons located at differing 'ability' (i.e., activity impairment). The divergence of each item from the Rasch model was assessed using the individual item fit Χ^2 ^statistic and residual (a residual >3.0 is generally taken to indicate misfit and <-3.0 that the item fits the model too closely), with item thresholds used to assessed whether, as a person's impairment increases, the probability of a maximum score on the item increases. The person-item threshold map was used to assess whether the questionnaire items represent respondents of all levels of impairment, and item characteristic curves whether any item significantly over- or under-estimates the impairment level.

### Internal consistency reliability

Cronbach's alpha statistics were calculated to assess the internal consistency reliability of the AIA scores.

### Convergent validity

The convergent validity of the AIA was assessed using Spearman's correlation coefficients between the AIA score and similar individual symptom and domain scores of the KHQ. The AIA score was expected to be significantly associated with the 'role limitations', 'social limitations', and 'physical limitations' domain scores of the KHQ.

### Divergent validity

The divergent validity of the AIA was assessed using Spearman's correlation coefficients between the AIA score and dissimilar individual symptom and domain scores on the KHQ. Because short-term UTI is not expected to have a major impact on one's personal relationships, divergent validity was assessed by calculating the Spearman correlation coefficient between the AIA score and the KHQ 'Personal Relationships' domain.

### Discriminant validity

The discriminant validity of the AIA was assessed by using Kruskal-Wallis analysis of variance to compare AIA domain scores (at first administration) between the initial clinical ratings for dysuria, frequency, urgency, suprapubic pain, and gross haematuria. It was hypothesised that AIA scores would differ significantly between the clinical groups.

### Responsiveness

It was hypothesised that the change in AIA score from visit 1 to the time when symptoms were no longer present would be related to improvements in the clinical evaluation of UTI. Change scores for the AIA and clinical evaluations were calculated by subtracting the TOC scores from the initial scores. Spearman correlation coefficients were used to assess the degree of association between the measures of change and Kruskal-Wallis analysis of variance for comparisons of group mean change scores.

## Results

### Patient characteristics

Two hundred and seventy six women were recruited to the study. The mean (SD) age of the women was 33.0 (11.46) years (range 18–78 years). Although the sample was ethnically diverse [12% (n = 34) black, 4% (n = 12) American Indian, 2% (n = 5) Hispanic], the majority of the women (70%, n = 193) in the study were white. Thirty-two women (12%) did not give any details of their ethnic origin. Five women (1.8%) reported that they had attended grade school only, while around half of the women (52.4%, n = 143) completed high school. A further 40.3% (n = 110) attended college and 15 (5.5%) attended graduate school. In response to the question 'which best describes what you were doing in the past 6 months', 49.5% (n = 135) reported that they had more than one role. One hundred and seventy five women (64.5%) were working full time, 65 (23.8%) were working part time, while 107 (39.2%) women were looking after the house and/or children full time. Fifty-three (19.4%) women were studying at university either full or part time, and 46 (6.8%) reported that they were engaged in some other role.

The majority of women were considered to have moderate or severe dysuria (n = 208, 75.4%), frequency (n = 238, 86.2%), and urgency (n = 237, 85.9%) at baseline clinical evaluation. Suprapubic pain was less common, with the majority of women (n = 179, 64.9%) rated as having either mild or moderate subrapubic pain. Haematuria was reported as absent in 151 women (54.7%).

### Factor structure of the AIA

On unforced principal components factor analysis on the AIA scores at baseline, one component was extracted that explained 78.6% of the variance in the data (Table 1). The loadings for each item on the single component were = 0.85. This supported the use of the AIA items as a single scale (called the AIA total score), scored as the sum of the 5 individual item scores.

### Data distribution

Patients used the full range of responses for each AIA item, with the median response for each item being 2.0 ('some of the time') (Table 1). The full range of scores was also used for the overall scale, with scores ranging from 0 (no limitations) to 20 (maximum level of limitations). Twenty-six patients (9.4%) reported no limitations at baseline with the distribution at lower levels of limitation appearing fairly uniform, but with smaller numbers of patients reporting extreme degrees of limitation (kurtosis = -0.98, SE = 0.29). The mean (SD) AIA total score was 7.56 (5.22) with a median of 7.00, also indicating non-Normality which was confirmed on Kolmogorov-Smirnov test (P < .001).

### Association of AIA score with demographic variables

There were no statistically significant associations between AIA scores and respondent age, ethnic group, or work status.

### Item functioning

Because factor analysis showed that the AIA is a uni-dimensional scale the one-parameter Rasch model was fitted to the data. Although the model fitted the data well (item-trait interaction Χ^2 ^= 20.32, P = .16), there was some suggestion of a small amount of misfit of the items and persons to the model (item-person interaction mean = -0.64, SD = 1.42). However, in terms of individual item fit, no Χ^2 ^statistic reached statistical significance and no residual was >|3| (Table 2). In addition, all items displayed ordered thresholds. The person-item threshold distribution map showed that the item thresholds cover the majority of respondent 'abilities' (i.e., activity impairment) in the sample. Only four thresholds were positioned at the same severity level, suggesting that all the items and response options are valuable in measuring the full range of activity impairment levels. Finally, the item characteristic curves demonstrated that the AIA performs very well across all activity impairment severity levels and does not significantly overestimate or underestimate any group's activity level.

**Table 2:** Individual item fit for the AIA items

**AIA Item**	**Residual Score**	**Chi-square**	**P-value**
1. Cut down on the amount of time you spent on work or other activities	1.735	6.899	0.075
2. Accomplished less than you would like	-1.789	3.356	0.340
3. Were limited in the kind of work or other activities	-2.118	3.827	0.281
4. Had difficulty performing work or other activities (for example, it took extra effort)	-0.652	0.545	0.909
5. Interfered with your social activities (like visiting friends, relatives, etc.)	2.577	5.696	0.127

### Internal consistency

The Cronbach's alpha coefficient for the AIA scale score was 0.93, confirming the internal consistency of the 5-item AIA. All items contributed equally to the reliability of the scale as all 'alpha if item deleted' coefficients were between 0.90 and 0.92.

### Convergent and divergent validity

The convergent validity of the AIA was demonstrated by high and statistically significant correlations between the AIA score and the KHQ 'role limitations' (r_s _= 0.76, P < .001), 'social limitiations' (r_s _= 0.71, P < .001), and 'physical limitations' (r_s _= 0.73, P < .001) domains. Divergent validity was demonstrated by a low and non-significant correlation between the AIA score and the KHQ 'personal relationships' domain (r_s _= 0.08, P = .201).

### Discriminant validity

The discriminant validity of the AIA was demonstrated by statistically significant differences in AIA scores between the clinical evaluations of dysuria, frequency, urgency, and suprapubic pain (Table 3). In particular, the greater the clinical severity, the greater the AIA score (P < .001). No significant difference in AIA score was found between the clinical evaluations of haematuria.

**Table 3:** Discriminant validity of the AIA – mean scores by clinical evaluation of 5 symptoms

**Symptom**	**Mean AIA total score by clinical evaluation**				**Χ**^2^
		
	**None**	**Mild^a^**	**Moderate^b^**	**Severe^c^**	
Dysuria	3.00	5.97	7.19	10.23***	22.04***
Frequency	3.71	3.03	7.50***	9.32**	38.96***
Urgency	3.50	4.37	6.88*	9.68***	37.39***
Suprapubic pain	6.24	6.39	7.97	10.70**	22.46***
Gross hematuria	6.96	7.49	8.26	9.59	7.07

Mann Whitney U tests used to test differences between clinical evaluations ^a^None and Mild, ^b^Mild and Moderate, ^C^Moderate and Severe *p < 0.05, **p < 0.01, ***p < 0.001

### Responsiveness

The AIA score was responsive to changes in the clinical evaluation of 'Dysuria' (r_s _= .28, P < .001), 'Frequency' (r_s _= .31, P < .001), 'Urgency' (r_s _= .31, P < .001), 'Suprapubic pain' (r_s _= .27, P < .001), and 'Gross Haematuria' (r_s _= .16, P = .008). In terms of mean AIA change by degree of improvement in clinical evaluation, clear trends were evident for increase in AIA improvement with increased degree of clinical evaluation improvement (Table 4).

**Table 4:** Mean (SD) AIA change scores by degree of symptom change

**Clinical Evaluation**				
**Symptom**	**Change score (baseline to final TOC visit)**^a^	**N**	**Mean (SD)**	**P-value**
Dysuria	0, 1	76	-5.32 (4.59)	<.001
	2	138	-6.53 (4.95)	
	3	53	-10.08 (5.46)	
Frequency	-1, 0, 1	48	-3.75 (3.73)	<.001
	2	132	-6.84 (4.79)	
	3	87	-8.69 (5.72)	
Urgency	0, 1	51	-4.67 (4.03)	<.001
	2	129	-6.34 (5.18)	
	3	87	-9.00 (5.18)	
Suprapubic pain	-2, -1, 0	54	-5.30(5.24)	<.001
	1	86	-6.09 (4.75)	
	2	89	-7.19 (5.18)	
	3	38	-10.24 (4.84)	
Gross hematuria	0	145	-6.19 (4.86)	.003
	1	54	-7.22 (5.46)	
	2	38	-7.08 (5.49)	
	3	30	-9.40 (5.48)	

^a^positive score = improvement; negative score = deterioration

## Discussion

This study has reported on the validation of the Activity Impairment Assessment (AIA) questionnaire in measuring the activity impairment associated with uncomplicated urinary tract infection (uUTI).

Strengths of the study include the fact that the validation was conducted on a large sample of 276 ethnically and socio-economically diverse women with uUTI in the context of a (non-comparative) clinical trial. The trial was specifically designed for the validation of the new instrument and thus incorporated a validated measure (the KHQ) as well as a clinical evaluation of symptoms.

On unforced principal components factor analysis, one component explained a high percentage (79%) of the variability in the AIA data. This component was named the AIA total score and was calculated as the sum of the five individual AIA items. The total score was found to have good internal consistency (Cronbach alpha = 0.93). However, it must be noted that Cronbach alpha scores >0.90 may indicate that items are too similar [20,21].

Mean scores for each AIA item indicated that feeling a lack of accomplishment and reducing time at work were the most frequently reported impairments in uUTI; interference with social activity was least commonly reported. The scores were significantly non-Normally distributed indicating that non-parametric tests should be used in the analysis of the AIA.

The AIA total score was found to have excellent psychometric properties. Convergent validity was indicated by high and statistically significant correlations between the AIA score and related KHQ items and domains. Divergent validity was indicated by the small and non-significant correlation between the AIA score and the (unrelated) KHQ 'personal relationships' domain.

The AIA total score displayed excellent discriminant validity in relation to clinical evaluation. It discriminated well between all clinical evaluation groups with the exception of the 'gross haematuria' evaluation, which might be expected to be less strongly related to UTI impairment. The AIA total score was also found to have very high levels of responsiveness, with strong associations existing between changes in AIA score and changes in clinical evaluation at TOC visit. These associations were all in the hypothesised direction and were again less associated with the 'gross haematuria' evaluation.

## Conclusion

The AIA questionnaire enables the assessment of the amount of time an individual's work or regular activities have been impaired as a result of their UTI. The questionnaire is composed of one domain, a total score, which has been found to have excellent psychometric properties. While the measure was designed specifically for use in a clinical setting, it is likely that it will also be suitable for use in an epidemiological context and beyond. Furthermore, although the AIA is validated here in the context of UTI it could also easily be adapted and used to assess activity impairment in other disease areas.

## Authors' contributions

DJW drafted the manuscript. DJC performed the statistical analysis. KK and KG participated in the study's design and coordination and helped draft the manuscript.
